# Supplementary material for: Genome-Wide Identification and Spatial Expression Analysis of Histone Modification Gene Families in the Rubber Dandelion Taraxacum kok-saghyz
Source: Plants (Basel). 2022 Aug 9;11(16):2077. doi: 10.3390/plants11162077 (PMC9415798; doi:10.3390/plants11162077)
Supplement: Supplementary file 1 [file plants-11-02077-s001.zip › Figura S1 S2 S4 S6 S7.pdf]

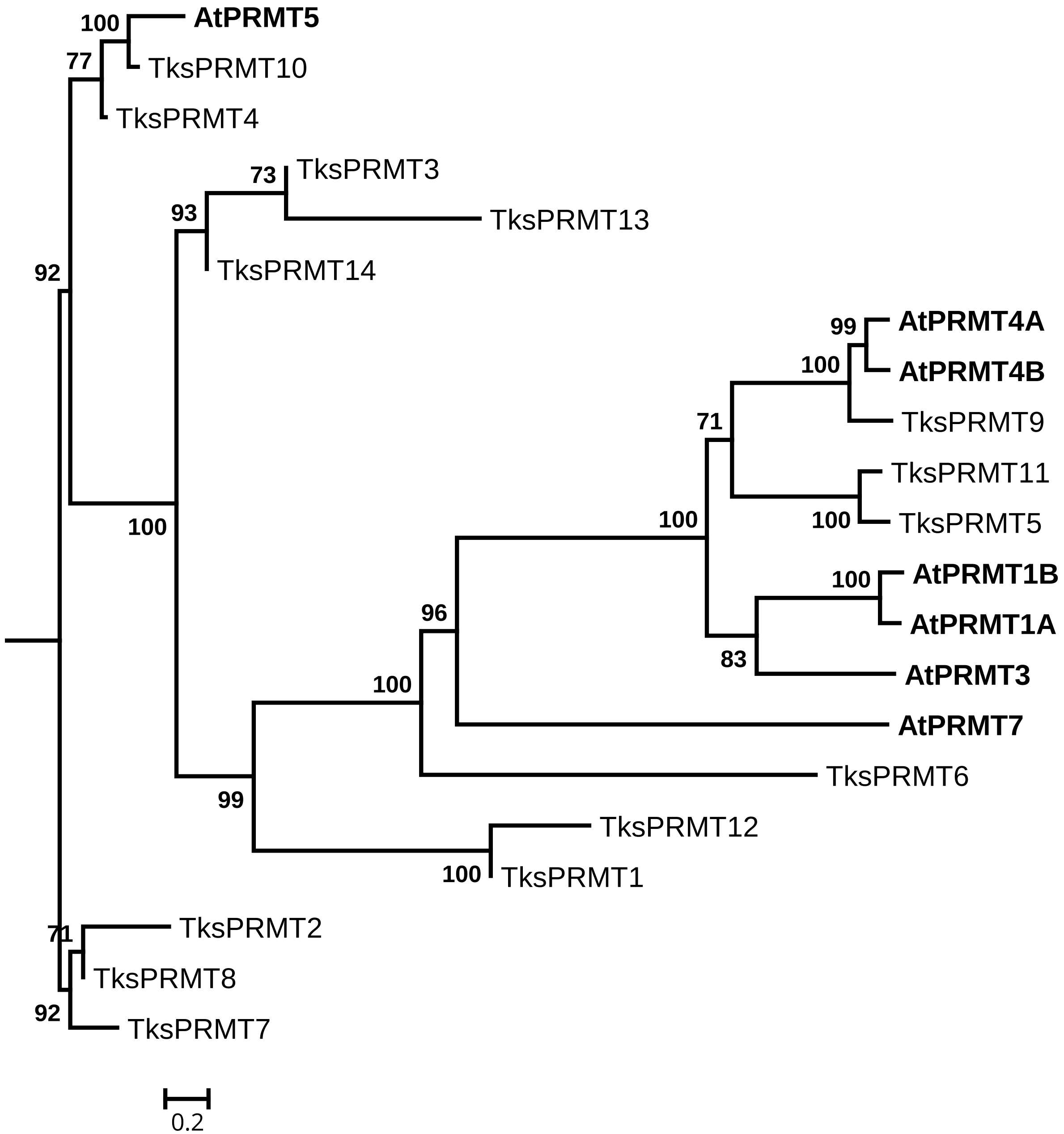

Figure S1. Phylogenetic tree of PRMT proteins of *Taraxacum kok-saghyz* and Arabidopsis (in bold). The numbers near the tree branches represent bootstrap values.

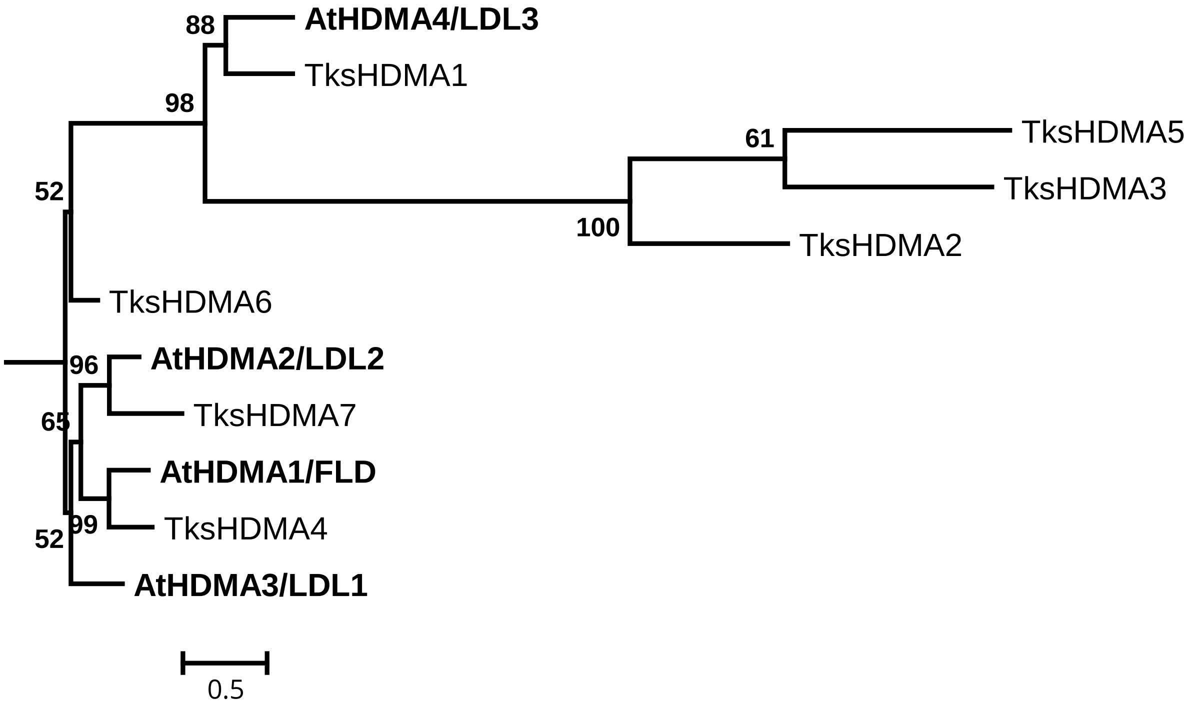

Figure S2. Phylogenetic tree of HDMA proteins of *Taraxacum kok-saghyz* and Arabidopsis (in bold). The numbers near the tree branches represent bootstrap values.

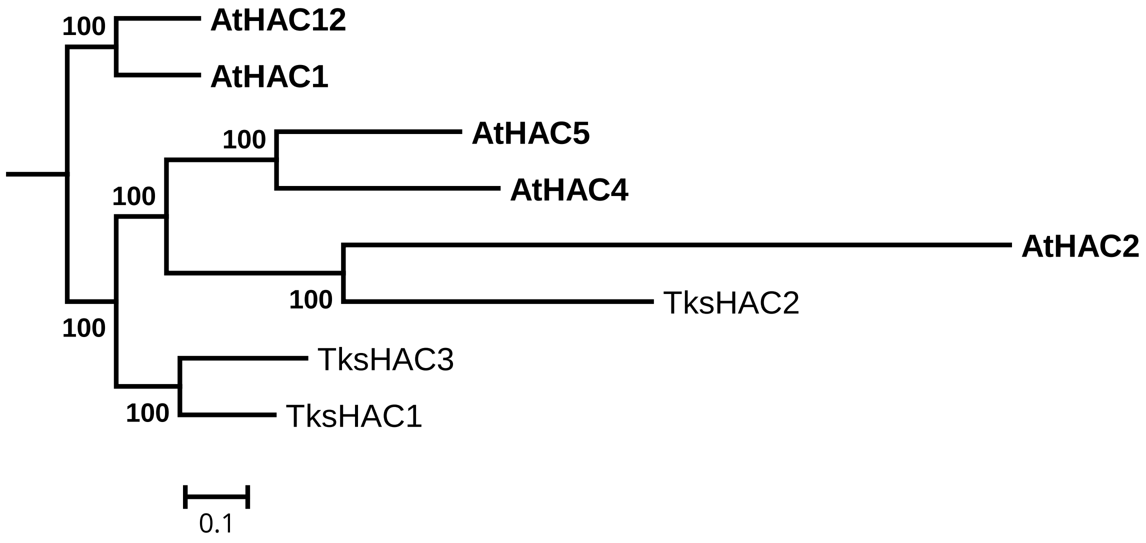

Figure S4. Phylogenetic tree of HAC proteins of *Taraxacum kok-saghyz* and Arabidopsis (in bold). The numbers near the tree branches represent bootstrap values.

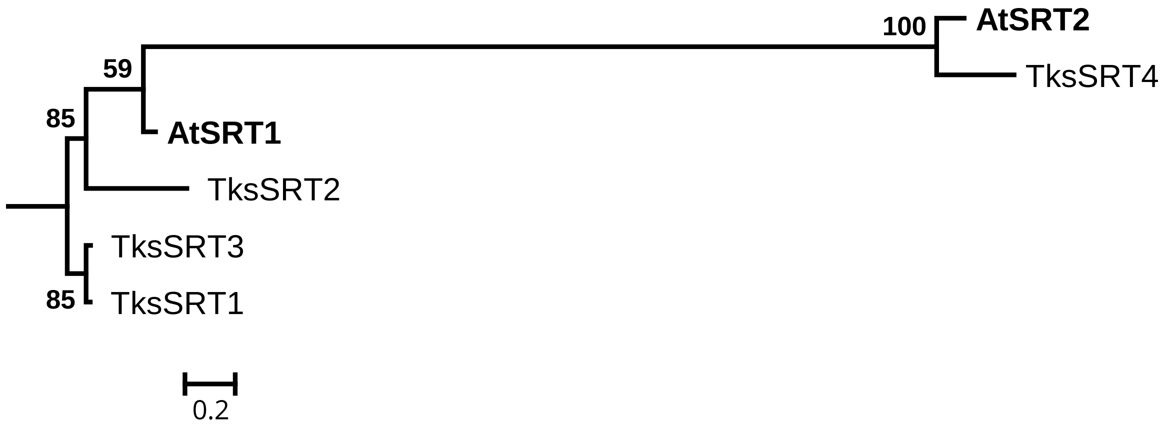

Figure S6. Phylogenetic tree of SRT proteins of *Taraxacum kok-saghyz* and Arabidopsis (in bold). The numbers near the tree branches represent bootstrap values.

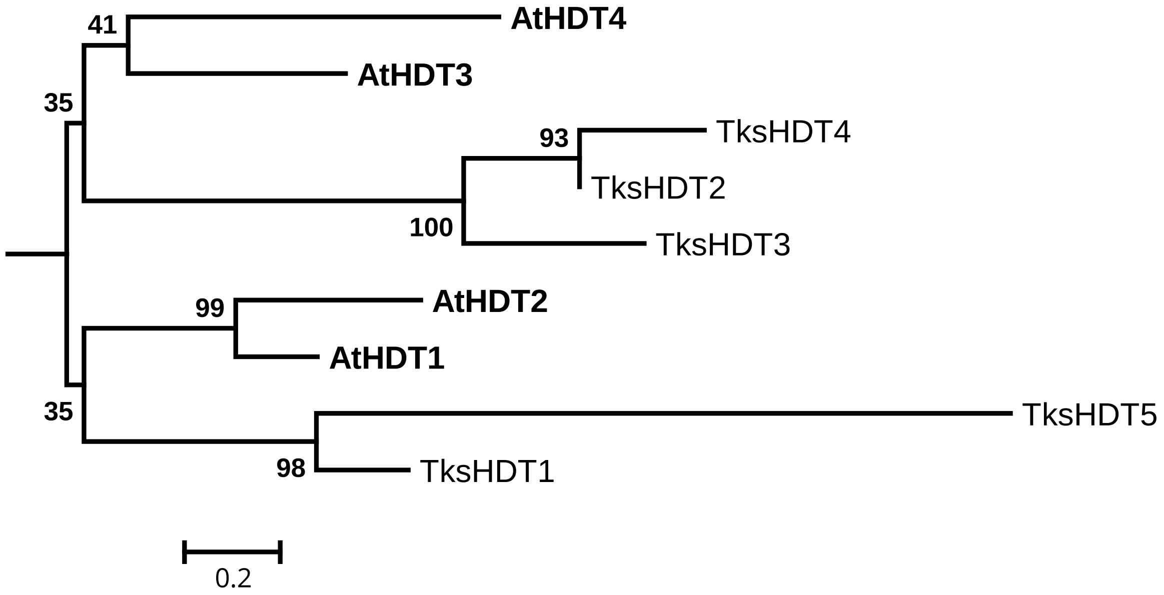

Figure S7. Phylogenetic tree of HDT proteins of *Taraxacum kok-saghyz* and Arabidopsis (in bold). The numbers near the tree branches represent bootstrap values.
