# Supplementary material for: Genome-Wide Identification and Spatial Expression Analysis of Histone Modification Gene Families in the Rubber Dandelion Taraxacum kok-saghyz
Source: Plants (Basel). 2022 Aug 9;11(16):2077. doi: 10.3390/plants11162077 (PMC9415798; doi:10.3390/plants11162077)
Supplement: Supplementary file 1 [file plants-11-02077-s001.zip › Figure S5 .pdf]

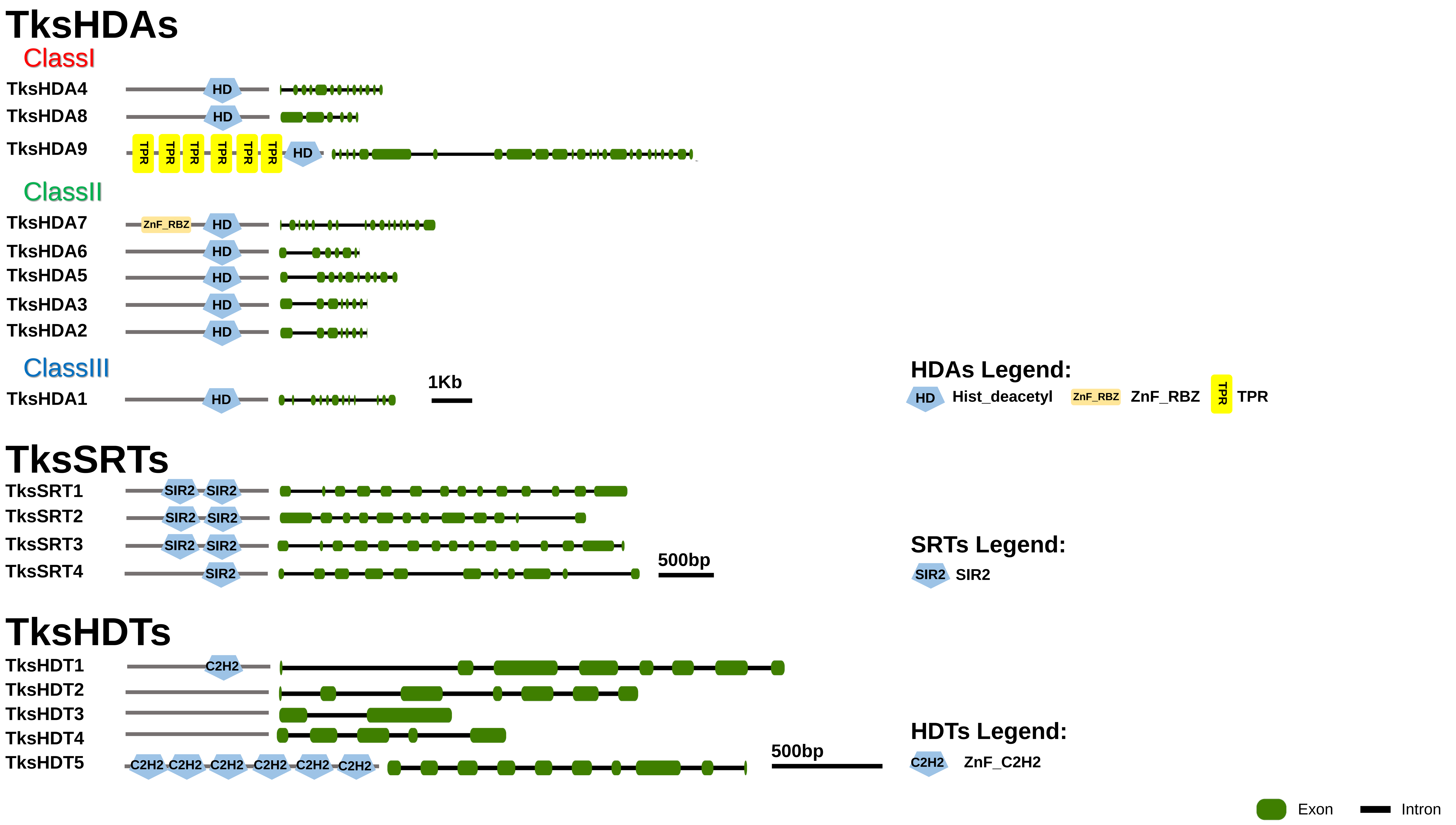

Figure S5. Domain composition and intron-exon structure of *Taraxacum kok-saghyz* HDAs, SRTs and HDTs.
