# Supplementary material for: Genome-Wide Identification and Spatial Expression Analysis of Histone Modification Gene Families in the Rubber Dandelion Taraxacum kok-saghyz
Source: Plants (Basel). 2022 Aug 9;11(16):2077. doi: 10.3390/plants11162077 (PMC9415798; doi:10.3390/plants11162077)
Supplement: Supplementary file 1 [file plants-11-02077-s001.zip › Table S4.pdf]

Tukey's pairwise test (p<0,05)

TksHAG10	DL	FL	RT	R
DL		0,112	0,6059	0,01427
FL	3,72		0,5529	0,4877
RT	1,792	1,928		0,0824
R	5,821	2,1	4,028	

TksHDA8	DL	FL	RT	R
DL		0,8504	0,8182	0,01078
FL	1,138		0,9986	0,02837
RT	1,236	0,2182		0,05729
R	6,45	5,312	4,533	

TksHDA7	DL	FL	RT	R
DL		0,7856	0,3487	0,001569
FL	1,329		0,7769	0,003967
RT	2,542	1,353		0,02263
R	9,069	7,74	5,569	

TksHDA9	DL	FL	RT	R
DL		0,9989	0,859	0,4204
FL	0,199		0,7921	0,3564
RT	1,111	1,31		0,8371
R	2,291	2,49	1,179	

TksHDMA4	DL	FL	RT	R
DL		0,9858	0,9765	1
FL	0,4775		0,8784	0,9848
RT	0,5691	1,047		0,9778
R	0,01127	0,4888	0,5578	

TksHDMA6	DL	FL	RT	R
DL		0,985	0,9304	0,8399
FL	0,4864		0,7852	0,6593
RT	0,8431	1,329		0,9952
R	1,171	1,657	0,328	

TksJMJ14	DL	FL	RT	R
DL		0,9889	0,9998	0,7735
FL	0,4377		0,9953	0,6032
RT	0,1106	0,3271		0,7319
R	1,361	1,799	1,472	

TksJMJ23	DL	FL	RT	R
DL		1	0,9978	0,8803
FL	0,0407		0,9987	0,8918
RT	0,2531	0,2124		0,9422
R	1,04	0,9991	0,7868	

TksJMJ25	DL	FL	RT	R
DL		0,2191	0,9996	0,9999
FL	3,078		0,3282	0,2348
RT	0,1388	2,614		1
R	0,07725	3,001	0,06966	

TksPRMT9	DL	FL	RT	R
DL		0,4005	0,8191	0,2253
FL	2,35		0,8571	0,9659
RT	1,233	1,117		0,616
R	3	0,6495	1,767	

TksSDG3	DL	FL	RT	R
DL		0,7528	0,7174	0,01543
FL	1,417		0,2412	0,06152
RT	1,509	2,927		0,003977
R	5,738	4,321	7,247	

TksSDG4	DL	FL	RT	R
DL		0,8551	0,8816	0,01543
FL	1,124		0,4663	0,04596
RT	1,036	2,159		0,005998
R	5,737	4,614	6,773	

TksSDG24	DL	FL	RT	R
DL		0,1155	0,1259	0,002417
FL	3,689		0,9999	0,0725
RT	3,602	0,08761		0,06642
R	7,846	4,156	4,244	
